# Identifying Ethical Issues in Mental Health Research with Minors Adolescents: Results of a Delphi Study 

**DOI:** 10.3390/ijerph13050489

**Published:** 2016-05-11

**Authors:** Elisabeta Ioana Hiriscau, Nicola Stingelin-Giles, Danuta Wasserman, Stella Reiter-Theil

**Affiliations:** 1 Department of Clinical Ethics, Psychiatric Hospital of the University Basel, University Hospital Basel, University of Basel, Wilhelm Klein-Str. 27, Basel CH-4012, Switzerland; Nicola.Stingelin@unibas.ch (N.S.-G.); s.reiter-theil@unibas.ch (S.R.-T.); 2 Department of Nursing, University of Medicine and Pharmacy “Iuliu Hatieganu” Cluj-Napoca, Str. Louis Pasteur nr 4, et 1, Cluj-Napoca 400349, Romania; 3 National Center for Suicide Research and Prevention of Mental lll-Health (NASP)/WHO Collaborating Center for Research, Methods Development and Training in Suicide Prevention, Karolinska Institute, Stockholm SE-171 77, Sweden; Danuta.Wasserman@ki.se

**Keywords:** assent/consent of minor, capacity to consent, confidentiality, Delphi, research ethics, risk of harm, sensitive topics, SEYLE (Saving and Empowering Young Lives in Europe)

## Abstract

Research with minors, especially for preventive purposes, e.g., suicide prevention, investigating risk or self-destructive behaviors such as deviance, drug abuse, or suicidal behavior, is ethically sensitive. We present a Delphi study exploring the ethical implications of the needs formulated by researchers in an international pre-conference who would benefit from ethics support and guidance in conducting Mental Health Research with minors. The resulting List of Ethical Issues (LEI) was submitted to a 2-rounds Delphi process via the Internet, including 34 multidisciplinary experts. In the first round, the experts reviewed the LEI and completed a questionnaire. Results from this round were analyzed and grouped in nine categories comprising 40 items. In the second round, the experts had to agree/disagree with the needs expressed in the LEI leading to a final list of 25 ethical issues considered relevant for Mental Health Research with minors such as: confidentiality of the sensitive data, competence for consenting alone and risk of harm and stigma related to the methodology used in research. It was shown that studies like SEYLE (Saving and Empowering Young Lives in Europe) trigger among researchers wishes to obtain specific recommendations helping to comply with standards for good practice in conducting research with minors.

## 1. Introduction

The current state of ethical guidance regarding Mental Health Research (MHR) with children and adolescents is a complex matter. On the one hand, ethically justified prevention research, especially in Mental Health with children and adolescents, is based on the objective to reduce the burden that mental illness places on young people, their families, and society. The necessity of conducting MHR is actively supported by the World Health Organization (WHO) being motivated by the alarming growth rate of risk-taking behaviors in teenagers [1]. On the other hand, this assertion appears to be important given the numerous prerequisites that have to be fulfilled before including children in studies may be permitted. Restrictions are particularly evident in research investigating sexual practices, deviance, drug abuse, alcohol consumption or suicidal behavior, which is considered to be sensitive although no consensus currently exists regarding what *sensitive research* means [2]. According to one understanding, a *sensitive* topic is one that is “intimate, discreditable or incriminating” [3]. Research touching sensitive issues such as illicit drugs, sexuality, or violence poses specific challenges to research ethics such as management of confidentiality of the sensitive data (e.g., the researcher’s practical dilemma of whether or not to inform the minor’s parents if information is given about behavior that brings risks of mental and/or physical harm) or the minor’s capability to consent alone for participating in the study [4,5].

From this background, it could be expected that researchers in the field are most familiar not only with formal, written ethical guidance such as codes or declarations. Moreover, they should be able to rely on advanced personal awareness and discourse of ethical issues helping them to tackle related problems in their research practice. However, there is a gap between theory and practice or the ideal and the reality of research as experience shows. Research teams are not always prepared to acknowledge upcoming topics as ethical issues that deserve being addressed. Leadership and commitment are required to make way for openness and reflection.

This paper relates to the SEYLE Study (Saving and Empowering Young Lives in Europe (SEYLE): Promoting health through the prevention of risk-taking and self-destructive behaviors, as a reference project illustrating sensitive MHR with minors and to an international pre-conference on mental health research ethics (“(When) Theory meets Practice—Ethical Issues in Research with Minors and other Vulnerable Groups” held under the auspices of the Child and Adolescent Psychiatry, 14 February 2012, Basel, Switzerland) exploring the ethical challenges and the researchers’ articulated needs for ethics support and guidance in research with children and adolescents.

The SEYLE project is supported through Coordination Theme 1 (Health) of the European Union Seventh Framework Program (FP7), Grant agreement nr HEALTH-F2-2009-223091. The authors were independent of the funders in all aspects of study design, data analysis, and writing of this manuscript. The Project Leader and Coordinator of the SEYLE project is Professor in Psychiatry and Suicidology Danuta Wasserman, Karolinska Institute (KI), Head of the National Center for Suicide Research and Prevention of Mental Ill-Health and Suicide (NASP), at KI, Stockholm, Sweden. Other members of the Executive Committee are Professor Marco Sarchiapone, Department of Health Sciences, University of Molise, Campobasso, Italy; Vladimir Carli, National Center for Suicide Research and Prevention of Mental Ill-Health (NASP), Karolinska Institute, Stockholm, Sweden; Professor Christina W. Hoven and Anthropologist Camilla Wasserman, Department of Child and Adolescent Psychiatry, New York State Psychiatric Institute, Columbia University, New York, USA. The SEYLE Consortium comprises of centers in 12 European countries. Site leaders for each respective center and country are: Danuta Wasserman (NASP, Karolinska Institute, Sweden, Coordinating Center), Christian Haring (University for Medical Information Technology, Austria), Airi Varnik (Estonian Swedish Mental Health and Suicidology Institute, Estonia), Jean-Pierre Kahn (University of Nancy, France), Romuald Brunner (University of Heidelberg, Germany), Judit Balazs (Vadaskert Child and Adolescent Psychiatric Hospital, Hungary), Paul Corcoran (National Suicide Research Foundation, Ireland), Alan Apter (Schneider Children’s Medical Center of Israel, Tel-Aviv University, Tel Aviv, Israel), Marco Sarchiapone (University of Molise, Italy), Doina Cosman (Iuliu Hatieganu University of Medicine and Pharmacy, Romania), Vita Postuvan (University of Primorska, Slovenia) and Julio Bobes (University of Oviedo, Spain). 

The SEYLE project illustrates how such personal awareness and discourse of ethical issues were strengthened in a pre-conference and subsequent Delphi-study including active MHR researchers.

SEYLE was a European FP7 Project, a multi-site randomized control trial (RCT) of interventions to promote mental health and prevent risk behaviors and suicide in European schools. The general objectives of the study were:

- to generate an epidemiological database on the general health status of European adolescents in the age group between 14 and 16 from 10 European countries (based on the collection of assessment data including demographic information, psychopathology, lifestyles, values and risk-behaviors)
- to evaluate three types of school-based interventions in comparison to a minimal intervention control group on mental literacy; these interventions included gatekeeper training (QPR), training adolescents in mental health promotion (Awareness) which is now named Youth Aware of Mental Health (YAM), and professional screening of adolescents for mental health problems and risk behaviors (ProfScreen).

The ethical issues identified in SEYLE included the following: Do the adolescents face any risks of harm caused by their participation? What kind of risks would they be? Are the risks specific to the research content? How will adolescents in need of emergency psychiatric help be identified and cared for during the study? How will the sensitive information obtained from the adolescents be treated (this including responses of the adolescents to questions about illegal drug use, sexual activity, smoking, alcohol consumption and suicidal thoughts and behavior)? How should an adolescent’s refusal to participate in research be handled? Is it considered valid if the parents give consent for a child or adolescent to participate in research but the child or adolescent has refused? How will privacy, confidentiality, and related issues arising from the generation of an epidemiological database be handled? 

Ethical issues mirrored by the SEYLE project as well as an overview regarding whether and how these issues are covered by existing research ethics codes and guidelines [6], were addressed within the pre-conference held in Basel, 2012. One major point raised by the participants referred to the problems with existing regulations and ethical guidelines that do not address the issue of drug trials, and that children and adolescents with psychiatric disorders are different from the general pediatric populations [7]. Although most of the codes refer to “mentally disabled persons” as a vulnerable population and they recommend additional safeguards for the protection of rights and welfare of such vulnerable research participants, they fall far short of the specific considerations that children and adolescents with psychiatric disorders deserve and need.

The emphasis was laid on the lack of relevant ethical guidelines for research with psychiatric patient populations, particularly children and adolescents. As examples, some reference codes were subjected to a briefly analysis for checking the specific considerations regarding the conduct of the MHR involving children and adolescents. The findings suggested that there was a need for normative documents to address the ethical questions regarding confidentiality, capacity to consent, risk of harm that arise in research into the complex area of preventive mental health and the specific considerations regarding research on sensitive topics, including communication with the family. Summarizing, the shortcomings identified by the analysis of the ethics codes and guidelines revealed the use of a heterogeneous terminology to describe minors *i.e*., those under a legal age of competence to give consent, and they fail to distinguish according to a developmental perspective (e.g., CIOMS uses the term children, children over 13 years, emancipated or mature minors and also term of adolescents in specific studies [8], Code of Human Research BPS uses only the term children and children under 16 [9], Declaration of Helsinki mentions only vulnerable population or subject who is deemed to be incompetent [10]). The Codes provide just a few general recommendations regarding the management of confidentiality in research involving sensitive issues with minors. The content of the guidelines and codes do not deal with personally sensitive or critical data for the mental health of the participants. There is a lack of clarity in any circumstances in which the researcher might have an obligation to breach confidentiality by disclosing sensitive information with a minor deemed competent to consent; no information is given on justificatory criteria e.g., what kind of disclosed information justifies breaching confidentiality; does any information relevant or potentially relevant to physical, psychological and social harms suffice? There is no mention or recommendation about how the consequences for the minor or the family of breaching a promise made of confidentiality should be dealt with; there is a lack of clarity about the investigator’s responsibility in ensuring confidentiality towards minors regarding disclosure of sensitive data. The documents leave it open, when the disclosure of „sensitive “information might be an appropriate or necessary action in order to protect the adolescent from harm.

In this regard, suggestions were made by participants for clarifying and explicitly formulating a (preliminary) List of Ethical Issues (LEI) regarding possible ethical guidance requested for the conduct of MHR involving children and adolescents. This LEI was used as a starting point for engaging experts in a Delphi process.

Two research questions will be addressed in this paper:

1. What needs for ethical guidance do research experts articulate for conducting safe, knowledge-based and fruitful MHR with children and adolescents?
2. What specific ethical issues should define MHR, others than those mentioned by the research ethics codes and guidelines?

Firstly, we will present the summary and evaluation of the results from the pre-conference and the preliminary LEI referring to the researchers’ needs. Secondly, we will define the key concepts —capacity to consent, emancipated and mature minors, vulnerability. Thirdly, we will describe the Delphi design and process, its participants and findings. In conclusion, the implications of the results and the limits of the approach will be discussed.

## 2. Key Concepts—Capacity to Consent, Emancipated/Mature Minors, and Vulnerability

Exploring the ethical dimension of competence reveals that it cannot be studied without acknowledging the doctrine of informed consent, and, more recently, the rule of shared decision making in medicine and health care. “Decisional capacity” is a central concept in health care, law and ethics, being defined as the *ability* of health care subjects to make their own health care decisions. According to one interpretation, “decision making capacity is a clinical assessment of a patient’s ability to make specific health care decisions, whereas competency is a legal determination of the patient's ability to make his or her own decisions in general” [11]. The law defines competence in terms of capacity to undertake the relevant tasks. The competence is seen as both task-specific and context-dependent, so the question that should be addressed in case of a minor is whether s/he is capable to make a particular decision or not. Assessment should be made taking into account the child’s development, the factors influencing his/her decision, the child’s emotional state, cognitive development and ability to balance risks and benefits.

Determining competence becomes more complex, e.g., when the researcher has to assess the capacity of a minor to participate in a sensitive study that refuses the involvement of her/his parents.

Although minors have generally been viewed as being incapable of making decisions, some groups of minors were allowed by law to consent to certain medical services, independent of their parents’ consent. Legally, a minor cannot give informed consent until s/he reaches 18—the age of legal majority [12]. In most countries, majority is reached with 18, but it may vary according to national legislation. Many ethics codes and guidelines recognize that adolescents have the emerging capacity to provide assent (in addition to their parents’ consent) to the extent to which their developmental capacity allows [8,10]. There are some exceptions to this position where a minor can alone give consent—*emancipated minors and mature minors*.

Regarding medical treatment there are circumstances where the law allows an ‘‘emancipated minor’’ to receive treatment without parental consent. This category of minors can give consent on the basis of their “status”—pregnant, married, enrolled in the army service, having children, living apart from parents, being independent financially, or on the “medical service” they are seeking (venereal disease treatment or HIV testing, contraception, prenatal care, abortion, mental health treatment, treatment for alcohol or drug abuse, after age 12).

The “Mature Minor Doctrine” represents a conceptual framework in which a minor can make treatment decisions without parental or guardian consent being required. The mature minor is allowed to consent alone for treatment in the following circumstances: the minor is an older adolescent (14 years or older) and is capable to give informed consent; the treatment will benefit the minor, it does not present a great risk for the minor and it is within established medical protocols [13].

In clinical practice in the UK, patients over the age of 16 years are permitted to give their consent to or to refuse treatment without parental involvement. In the case of patients under the age of 16, any decisions requiring consent are made by parents or guardians. However, some recognition has been given to those under 16 years of age; the concept of the Gillick competent child refers to the minors under 16 and who are deemed mature enough to understand the nature and implications of a clinical treatment or procedure [14]. The Gillick decision defines competence as the ability to understand information about the proposed treatment. This includes the treatment’s purpose, nature, likely effects and risks, chances of success and the availability of any alternatives, and the consequences of no treatment. The subjectivity of the concept arises because the law leaves the decision about whether a child is Gillick competent to the individual practitioner.

It is generally recognized that competency includes at least a factual understanding of the illness and treatment alternatives, including their risks and benefits, and the capacity for rational decision-making. Legal standards for decision-making capacity for consent to treatment vary somewhat across jurisdictions, but generally they embody the abilities to communicate a choice, to understand the relevant information, to appreciate the medical consequences of the situation, and to reason about treatment choices [15]. Most minor consent statutes require an understanding and appreciation of the nature and consequences of treatment alternatives. The MacCAT-T is the most fully developed standardized method of assessing competence, which closely follows the definitions of capacity as reflected in the common law of England and the United States [16].

Related to these aspects, vulnerability is a key-term used when referring to characteristics of different groups and their risk of being harmed as a consequence of their participation in research. Being at a higher risk to exploitation than healthy participants, vulnerable populations are entitled to special protection and assistance [8]. The factors identified as defining the minor’s degree of vulnerability include their legal inability to give consent, their dependency upon adults, the intertwined issues of limitations in their developmental stage [17,18], diminished autonomy, and limited cognitive abilities that do not allow children to comprehend the risk-benefit concept of research. For example, vulnerability will be high for any adolescent enrolled in sensitive research who is identified as being at risk due to drug use, a history of suicide attempts, or who is suffering from a mental health disorder (e.g., depression) if the confidentiality is not ensured. Disclosure of information concerning adolescents’ data to parents or authorities should therefore be given special attention; recalling the ethical principle of “do not harm”, breaching confidentiality towards minors by disclosing data promised by the researcher to be kept confidential, might harm the minor (socially, psychologically or physically) [19,20,21]. Therefore, when conducting research with such minors, the investigator should consider the cumulative medical, social and psychological consequences for each participant [22,23].

## 3. Materials and Methods

Briefly defined, the Delphi approach is a tool for expert problem solving. According to Linstone and Turoff [24], the Delphi approach is considered well suited for a consensus-building process concerning a specific topic by collecting data from a panel of experts with experience in specific areas. Using the Delphi method in research is supported by the need for a consensus-building communication process. It is based on gathering opinions from a panel of experts about the relative importance of an issue through an iterative process, taking place over a number of “rounds”, usually 2 or 3 (or more) over a relatively short period of time. Starting with the second round, the responses from the previous rounds are provided as feedback to the participants. They are allowed to reassess and change their initial judgments or positions regarding the topic discussed in previous rounds having considered the colleagues’ opinions [25]. In the last decades, Delphi method of systematically of gathering input from experts on a topic has been widely applied in education, economics and health [26]. The basic assumption underpinning its employing in research is supported by the need for a communication process with expert consensus.

**Figure 1:**
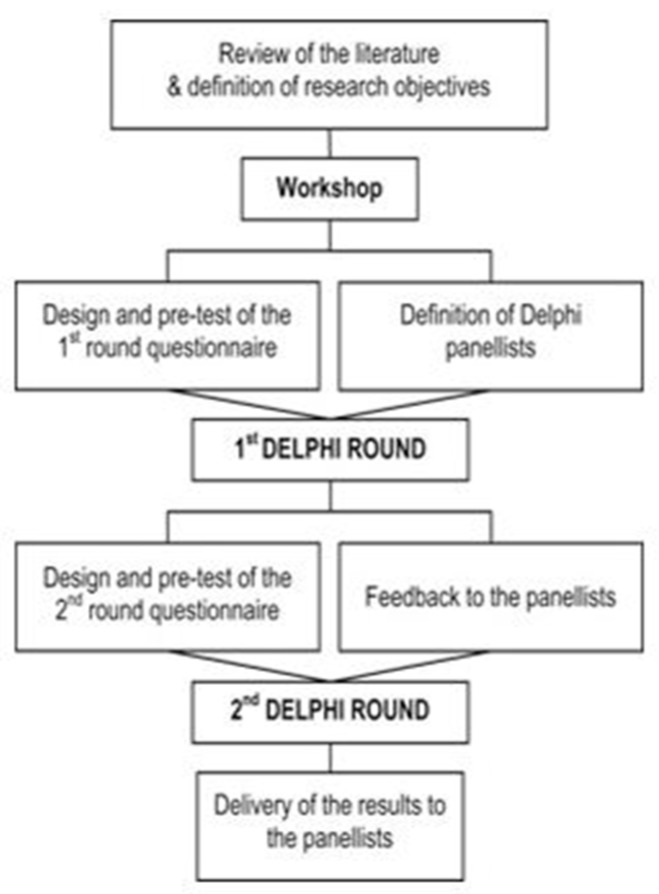
Delphi process (source model).

We designed and conducted a Delphi process via the Internet (see Figure 1). Figure 1 shows the source model that was adapted for designing and conducting the Delphi process [27] (Originally, the source model was proposed by a group of researchers for studying competition of the electricity market by using the Delphi technique).

**Figure 2:**
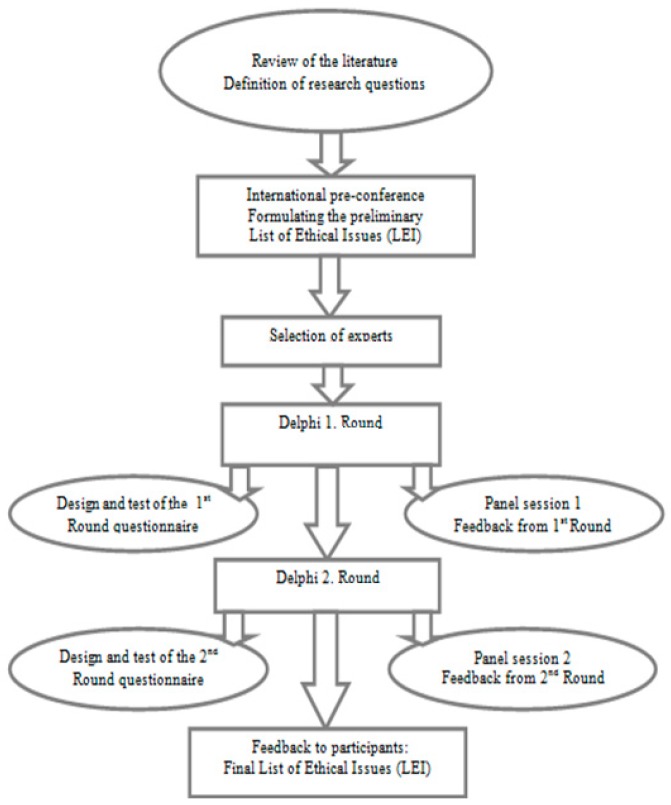
On-line Delphi process (adapted).

Figure 2 depicts the structure of the online Delphi process that we used consisting of the following stages: the pre-conference, selection of the experts, two rounds along with two panel sessions and feedback to participants after each round and final feedback.

Stages of the Delphi process:

- *Pre-conference*—formulating the preliminary LEI
- *Selection of Experts*—sending invitation letters for participation
- *1st. Delphi Round—*Design, distribution, completion of data collection and analysis of the first on-line questionnaire
- Panel session 1
- *Feedback to participants—*communicating the new LEI and summary of the results from the 1. Round
- *2nd. Delphi Round—*Design and distribution of the second on-line questionnaire
- Panel session 2
- Feedback to participants from the 2. Round
- *Final Feedback—*Providing final LEI to participants

### 3.1. Pre-conference—List of Ethical Issues (LEI)

Following the international pre-conference, a preliminary List of Ethical Issues (LEI) was formulated regarding the main ethical issues encountered in research with minors that would benefit from ethics support and guidance. When formulating the ethical issues, special attention was given to those themes that had triggered very intensive discussions among participants and had been perceived as ethically relevant for the practice of research with minors (see Table 1).

### 3.2. Selection of Experts—Using the SEYLE Study as a Paradigm Project

The group of experts invited to participate in the Delphi study included international researchers (principal investigators and researchers) involved in the European FP7 Project SEYLE [28,29]. SEYLE is used as paradigm project to illustrate ethical questions arising in the preparation, performance and analysis of MHR with adolescents that touches sensitive topics. Experts of mental health, researchers who participated in the international pre-conference, physicians and other health professionals, as well as experts of medical ethics were invited to take part in the Delphi process. Those who accepted the invitation were informed that their response to the first round was a prerequisite for participating in any subsequent round.

**Figure 3:**
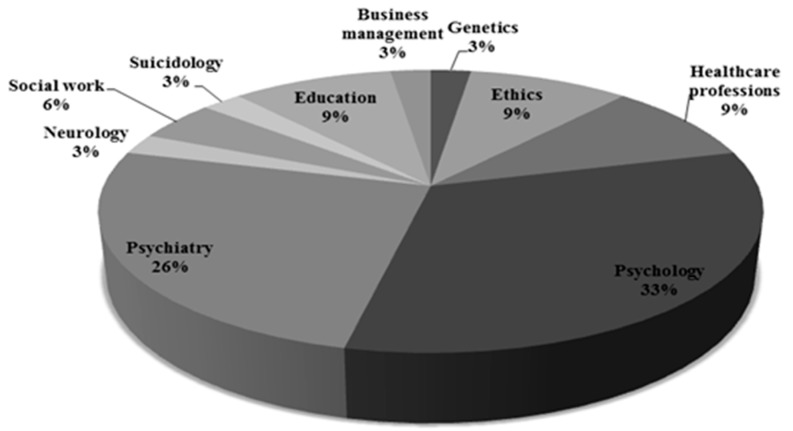
Interdisciplinary profile of participants (more than 50% of the Delphi participants had two or more qualifications).

We sent invitation letters to 60 experts. The resulting Delphi group included 34 experts who agreed to participate; their professional qualifications are shown in Figure 3.

Working with minors was included in the professional activity of 27 experts. As target groups were mentioned: handicapped children, children and adolescents with psychiatric disorders (focus on self-harm, suicidal behaviors, risk-behavior and personality disorders), preschoolers with Autism Spectrum Disorder (ASD), parents of children and adolescents with life-threatening illnesses, young adults. Seven experts reported no work directly related to minors. Forty-two percent of the experts reported involvement in studies with minors as Principal Investigators, 28% as Researchers, 14% as Clinical Research Associates, Research Manager (7%), or Ethical Advisor (5%). Among the studies undertaken with young people the experts mentioned: prevention studies (15), public health research (10), clinical trials (8), behavioral (7) and observational studies (6), and (rarely) physiological, genetic and mental health studies.

### 3.3. First Delphi Round

The first online questionnaire was made available to the experts in the first round. In the first part of the questionnaire, they were asked to approve of or add to or modify the preliminary LEI formulated within of the pre-conference. The questionnaire consisted of 5 Yes/No questions, 7 multiple choice questions, 2 single choice questions and 6 open questions. Additional explanations were requested. Responses were analyzed within panel session 1.

### 3.4. Panel Session 1 and Feedback to Participants

The role of this panel was to identify similar issues, to organize the data in broad categories and to prepare feedback to participants. 40 items were identified and organized into nine separate categories.

### 3.5. Second Delphi Round

The second online questionnaire was available to the experts in the second round. They were asked to agree or disagree with the listed ethical issues. The percent agreement was calculated for each item. As the literature suggests the investigators established a priori decision rules on the handling of rating information and the definition of consensus [30,31,32]. We established that each Issue that received more than 70% of the experts’ positive votes reached consensus; if less than 30% agreed, the Issue was dropped. The Issues that received less than 70%, but more than 30% of the experts’ positive votes were retained as “ambiguous” and put to discussion at panel session 2.

### 3.6. Panel Session 2 and Feedback to Participants from the 2. Round

The task of this panel was to analyze each ambiguous Issue and vote whether it would be modified, accepted or eliminated. In the end, the LEI was finalized, followed by feedback to participants about which ethical Issues had reached consensus.

### 3.7. Final Feedback

The final LEI was provided to all participants in the study.

The ethical approval for conducting the study was obtained from the Ethical Committee of North-West and Central Switzerland (18/09/2012). After reviewing the documents, the Ethical Committee was able to state that the conduction of the study is not ethically objectionable (*cf.* Article 51 paragraph 2 federal law of human research) and, thus, not requiring formal review. As the research project was clearly described in the cover letter, filling out the questionnaires by the respondents was regarded as their consent for participation in the study.

The whole Delphi process took place between October 2012 and May 2013 and was conducted using Google Docs, a web-based survey and data collection system.

## 4. Results

Thirty out of 34 experts approved of the preliminary LEI recognizing their needs therein. When they were asked to evaluate it, two suggestions for modification and eight for adding new items were put to discussion and analyzed in panel session 1. The panelists agreed on one new item to be added to the LEI and on rephrasing the items 3 and 5 (see Table 2).

### 4.1. Ethical Issues Encountered in Research with Minors

After reviewing the LEI, the experts identified the most frequent ethical issues encountered in researches they conducted such as the difficulty to obtain informed consent of a minor aged between 14 and 16 having the capacity to consent, and breaching confidentiality in risk-taking research with minors. Other issues mentioned were: problems with the minor’s refusal to participate if the parents gave their consent, risk of harm related to the methodology used in research, the management of critical data if the minor (considered to be at risk) decided to leave the study, handling of information with long-term impact or impact on family members, and giving feedback about research findings.

### 4.2. The Circumstances under which Parental Consent Might Be Waived

Exploring the circumstances under which parental consent might be waived more specifically, we grouped the experts’ responses as follows: circumstances under which the minor’s consent might suffice alone, and situations that may justify waiving parental consent. We identified six major themes also carrying normative weight:

- *benefit for the minor* (e.g., if parents withhold their consent to a study with potential major benefit for the minor and if supported by the decision of an institutional review board (IRB));
- *minimal risk/no harm* (*i.e.*, “if no harm is possible, e.g., questionnaire studies”; “if the research entails only very little risk and burden”; “if the research is more of a routine, with no significant risk or additional unusual tasks required from the participants”; “the research does not involve any procedure being performed which would not be part of routine case management”);
- *the nature or consequences of the research* (*i.e.*, “in certain sensitive topics if agreed by IRB”; “if the research involves intimate issues like sexual orientation—homosexuality, or the relationship between the minor and his/her parents”; “if the consequences do not affect the parents”);
- *parents’ status* (e.g., “if parents are not involved in the education of the child at present”; “if parents are not available“; “if parents lack competence for consenting“; “antisocial/problematic parents”);
- *minor’s status* (*i.e.*, “always, if the minor is deemed to be competent (from the age of 14))”;
- *special circumstances* (“life-death situation”).

### 4.3. Minor’s Dissent

As far as a minor’s dissent to participate was concerned, nine experts agreed that they had dealt with the issue in all studies they conducted; seven experts had not faced the issue in all of in their studies; 13 experts occasionally encountered the issue. Active parental consent (Opt-in procedure: the introductory letter explains the nature of the study and provides a method to document permission) was considered appropriate to be requested in sensitive research with minors by 23 experts; eleven experts opted for passive parental consent (Opt-out procedure involves distributing a letter to the children's parents or guardians explaining the nature of the study and providing a method to retract permission). In situations where a minor was considered competent to give consent, but refused his/her parents to be asked for consent, performing a competence test with the minor was suggested: if competent, s/he should be allowed to decide whether or not to enroll in the study.

### 4.4. Obligation to Disclose Sensitive Information

Regarding the question whether information should be reported to the parents if their child aged 16 had enrolled in a study on risk-taking behavior the experts’ opinions were grouped in two classes:

- *quantitative* (how much information): *i.e.*, “the overall results of the study”; “basic information on the scientific question(s) of the study”; “strictly the information necessary for taking the right measures in case of high risk of harm”, or “no results”;
- *qualitative* (what kind of information): *i.e.*, “if the minor shows behavior that puts himself/herself or others at serious risk (planned crimes)”; “if the child is reporting significant risk behaviors and health problems”; “suicidal ideation and behavior, pathological gambling, drug abuse, promiscuity”; “risk for suicide, severe alcohol or in case of life-threatening behavior (self-cutting, suicide attempt, bulimia, anorexia)”; “information about possibility of the need of extra-support (extra-care): medical or psychological”.

### 4.5. Breaching Confidentiality Agreement with a Minor Deemed Competent to Consent

With respect to the circumstances under which the researcher should breach the confidentiality agreement with a minor deemed competent to consent, according to the experts’ opinions reporting should take place,

- “if the participant is at risk of suicide (e.g., suicidal ideation or attempts)”;
- “if the physical or mental health of the minor is in serious danger (suicidal, self-damaging behavior, severe mental disorder, drug abuse, sexual abuse)”;
- low or non-compliance to a proposed intervention if the data showed a self-harm risk (e.g., s/he does not come to the therapy sessions);
- child abuse or pressing need of medical care.

### 4.6. Handling of Critical Data

Regarding the handling of critical data (Critical data refer in our study to self-damaging behavior), e.g., if the minor decided to withdraw from the study, most respondents agreed that the data must be deleted (if retractable). Besides this opinion, it was suggested that all data from the withdrawers should be separated from the active data and the active drop-outs should be analyzed. The use of critical data recorded before the time of withdrawal should be explained in the consent form. In case of an “active withdrawal” of the participant (e.g., written or oral), the data should be erased, but in case of passive withdrawal from the study (e.g., no show at follow up evaluation) data should be kept. It was also recommended that, if critical data raised the suspicion of any form of self-harm, measures should be applied to protect the minor even if the minor decided not to continue with the study.

### 4.7. Preventing Minors to Withdraw from the MHR Studies

Concerning this issue the experts’ recommendations focused on how to motivate minors to participate: e.g., by “providing interesting rewards for their participation”; “presenting the research in interesting yet honest way”; “to motivate them with certificate of attendance, small prizes”; “to offer them interesting workshops”; “to restrict feedback to parents”; “to encourage their feedback and ensure they are informed about results”. On the other hand, the experts suggested information about the research to be provided appropriately: e.g., “good explanation of the nature and the purpose of the study; the risks and benefits of participating in research”; “explanations concerning the value of the study and ensuring the anonymity of the respondents”; “using age appropriate language”. It was also recommended to keep “the study as short as possible”; “to provide appropriate safeguards or limits of confidentiality (especially in studies which can result in discrimination and social stigma), or “to follow best research standards e.g., CIOMS”.

In the second round, the experts’ agreement/disagreement with the needs was obtained. The questionnaire consisted of 40 items grouped in nine categories (Table 3):

Out of the 40 items, 25 reached consensus, one was rejected, 14 items did not reach consensus (neither for acceptance, nor for rejection) and were put to discussion in panel session 2. The panelists analyzed and voted on each ambiguous item about retaining or dropping it. All 14 ambiguous items were excluded from the final LEI (Appendix Table A1—List of Ethical Issues).

## 5. Discussion

In this study, a modified online Delphi process was used to build on the LEI referring to the needs considered relevant for researchers who conduct mental health (prevention) studies with minors. To our knowledge and according to a systematic literature review [33], *empirical* studies exploring ethical issues in MHR on sensitive topics with children and adolescents are scarce. The ethical issues identified by experts as being the most important in conducting MHR with minors adolescents were: problems with (1) the minor’s capacity to give consent and the circumstances under which parental consent might be waived, and (2) managing confidentiality in MHR with competent minors adolescents (see Table 3). “Harm” (or prevention of harm) does not show in Table 3; however, the topic was addressed thoroughly under the heading “circumstances under which parental consent might be waived “and it represents an important ethical issue. Thus, risk of harm and related content (3) will be discussed in more detail now.

### 5.1. Competence to Give Consent

With respect to the competence to give consent, particular interest was placed on exploring the circumstances under which the consent of a competent minor might suffice alone in MHR.

Looking at clinical practice, in UK, patients over 16 are permitted to give their consent to or refuse treatment. The children under 16 may be allowed to consent to medical procedures if they are deemed mature enough to understand the nature and implications of clinical treatment or procedure, can appreciate foreseeable consequences and make competent decisions in question, but they cannot refuse the treatment; this condition is being known as “Gillick competency” [34,35,36,37]. Despite relatively broad usage of “Gillick competency” in medical context it has been already claimed the inappropriateness of its application in research. Hence, applying this condition to research requires consideration of whether the minor has the capability to understand the nature of the research, his/her rights as a subject, and the risks and benefits of their participation in research.

In clinical research with minors, the doctrine of informed consent requires the approval of a legal representative of the child. It may be assumed that children over the age of 12 or 13 years are usually capable of understanding although such knowing is insufficient to permit participation in research unless it is supplemented by the permission of a parent, a legal guardian or other duly authorized representative [38,39]. Assent is the concept of providing agreement to participation in research where full consent is not possible. It is recommended that assent be sought for participation in research at an age appropriate level, and as suitable to the complexity of the project under consideration. Children should be involved in the decision to take part in research as their developmental capacity dictates. Most of the research ethics codes state that the minor’s dissent (the voicing of a desire not to take part in research) should be also respected. Fully developed articulation of the reasons for dissent should not be required to end a child’s participation in research, but the reasons for dissent should be explored to determine their validity [8]. Related to the topic of the minor’s dissent the participants in the Delphi study recognized the importance to respect the minor decision but most of them agreed on a discussion with the minor for clarifying the reasons for refusal. Seven participants declared they respected unconditionally the minor’s dissent in all their studies.

Regarding the minor’s consent many researchers argue that applying such category in sensitive MHR raises difficulties in practice [40]. Regarding the age when the adolescent can give consent for participating in research is still a lack of consensus in the legislation of many countries. For example, in Canada and Australia, children can give consent to participate in research at the same age with consenting to treatment, but only in the absence of serious risks to their health [41]. CIOMS includes a specific reference regarding the waiving of parental consent in studies that involve an investigation of adolescents` beliefs and sexual behavior or use of recreational drugs. According to CIOMS document, giving up parental consent is allowed if “parental knowledge of the subject matter may place the adolescents at some risk of questioning or even intimidation by their parents”. Some researchers argue that it may not be appropriate to ask for parental consent in some situations, namely in sensitive research with young people investigating sexual behavior [42] or drug use [43]. A suggestion made by American experts was not to seek parental consent when the involvement of parents might be detrimental to the interests of the adolescent [15]. The Royal College of Psychiatrists’ states that parental consent “is a necessary, but not a sufficient criterion for approval for a child to be included in research involving more than minimal risk” [44,45]. Here, the term ‘minimal risk’ is used to indicate the acceptable level of risk and defined as precisely the risk that is encountered in daily life or during the performance of routine physical or psychological examinations or tests.

Regarding the minor’s consent most of the participants in the Delphi study considered the issue difficult to put in practice, especially in research touching sensitive issues with adolescents aged between 14 and 16. Twenty participants agreed that the consent for a minor participating in research should be authorized *always* by the parents or the guardian or legal representative if parents are not available.

The minor can only give assent, consent from parents/representative being always needed, regardless the age of the minor or the research context. Nine participants agreed on the procedure for assessing the capacity of a minor to give consent.

Complex treatments and interventions, as well as the consequences of their application demand a higher level of understanding. In this respect, the assessment of a child’s capacity to consent to medical treatment requires special attention. In clinical context the practitioner is responsible, on the background of medical training, to deal with the assessment of the minor’s capacity if a treatment or procedure is proposed or required to be applied in minor’s case. Totally opposite is the situation of the researcher. The researcher, who is not (necessarily) clinician, is not trained and qualified to assess the capacity to consent of a minor aged under 16 as participant in research. One suggestion was to involve a psychologist with expertise in this area. In addition, by applying the principle of analogy, looking at current thinking on obtaining informed consent or assent applied to minors in medical practice, may help researchers consider the extent to which minors should be held as being autonomous regarding both their behavior and their participation in research, and then to balance this with the duty to protect.

### 5.2. Limits of Confidentiality

In a previous study, the issue of confidentiality was found to be a neglected topic in the guidelines [21]. Only few, general recommendations regarding confidentiality in sensitive research with minors are provided by some codes. They show a lack of clarity on any circumstances in which the researcher might have an obligation to breach confidentiality by disclosing sensitive information; no advice is given on justificatory criteria, e.g., what kind of disclosed information justifies or even requires breaching confidentiality. In particular, no criteria are provided that allow for the assessment of the type or degree of risk of harm that must be involved in order to justify or require the breaching of the promise to hold information confidential.

Regarding confidentiality, the central reason appreciated by most participants in the Delphi study to justifying or even requiring disclosure was if information provided by a minor, even under the understanding that it would be kept confidential), suggested that s/he might be at *risk of harm that could be prevented by disclosure*. A high risk of harmful behavior to him/herself (suicidal thoughts and ideation, sexual abuse) or to others, severe mental disorder, drug abuse or need for medical care were considered by the participants to our study the circumstances under which the duty to disclose is required.

From an ethical view, the researcher has, according to the principle of nonmaleficence (“do not harm”) an ethical and legal obligation to protect participants. Nonmaleficence carries special weight if the research participants are vulnerable like those with diminished autonomy. Thus, respecting confidentiality is a prima facie or conditional duty that can be overridden by other considerations of higher priority in a given situation. A primary justification of disclosure is obligation to reduce the risk of death. In assessing the risk the researcher must weigh the probability that harm will materialize and the magnitude of the anticipated harm against the obligation of maintaining confidentiality. In order to mediate between the important goals of protecting and avoiding harm, but not infringing other rights and duties by too readily disclosing sensitive information, a systematic criteria catalogue is needed of different risks of harms (physical, psychological and social). The catalogue should be graduated, starting at one end with serious harms that require that the researcher discloses the information (whatever the level of maturity may be). The other end of the catalogue contains minimal risks that can be contextually weighed–up, together with considering the level of maturity that a minor has reached, in deciding whether to disclose or not. Thus, for ‘minimum’ risks taken by a mature minor, confidentiality might prima facie prevail over arguments for disclosure.

### 5.3. Risk of Harm

Risk of harm deserves being explored *in some more details. S*ensitive research such as the SEYLE study has to reflect whether *participation as such mi*ght be harmful to the child or adolescent. For SEYLE safeguards were planned to guarantee that such risks would be detected and the affected participants released from participation and/or transferred to appropriate support [28]. In spite of scientific evidence there is a widely spread concern that exposure to suicide-related content will have iatrogenic effects. Specifically one ethical question was raised in SEYLE as a school-based preventive study of suicidal behaviors: could the methodology used be detrimental to participants? Looking at empirical data can help to answer this ethical concern. According to a study conducted by Gould *et al.* [46] which assessed the iatrogenic effects of a youth suicide screening program in a randomized controlled trial, the students exposed to suicide queries were no more likely to report suicidal ideation than unexposed students. The study also reported that high-risk *s*tudents with depression, substance use or previous suicide attempt in the intervention group were neither more suicidal nor distressed compared to the control group. In contrast, results showed a significant reduction of distress (*p* = 0.01) and suicidal behaviors (*p* = 0.02) in this group compared to high-risk students in the control group. Significant decrease by approximately 50% was observed in the outcome measures both in severe suicidal ideation and suicide attempts [47].

The lack of evidence supporting potential iatrogenic effects has been reported in several other intervention studies on suicidal behaviors [48]. The results from the SEYLE study were in line with those reported by Gould and colleagues, showing no evidence that indicated iatrogenic effects; moreover, the rate of severe suicide ideation and suicide attempts among adolescents significantly decreased. Based on the evidence resulting from the SEYLE study, it appears that implementing suicide prevention research as well as interventions in schools, do not pose a risk of harm among youth; rather, the interventions actually seem to have a positive effect in promoting mental health awareness and reducing suicidal behaviors [49].

## 6. Strengths and Limitations

Summing up and evaluating our Delphi study, it is worthy to note some considerations about its strengths and limitations. According to Miller [50] “the common surveys try to identify *what is*, whereas the Delphi technique attempts to address *what could/should be*”. In this regard, we consider the Delphi process as a suitable method for identifying *ethical* issues in research with minors requiring qualitative methodologies and an openness towards the dimension of what could/should be.

In our eyes, another advantage of the Delphi approach is the potential it holds to acknowledge the contribution of each participant. This type of study is supposed to facilitate a ‘fair’ representation of the views of each expert; this is important as the respective understanding may be significantly influenced by the particular area of expertise, experience or occupational position of the expert and, thus, enriching the results. Delphi is extremely sensitive to the composition of the group experts and the clarity of the questions.

The on-line application has proved to be cost-effective in term of time and expense, it avoids investing time for extended meetings, and it allows experts to participate over time, each round being assigned a time in which the participant is asked to respond. This way the experts can provide their input without time or group pressures or interpersonal influences.

Among the shortcomings of the Delphi method have to be mentioned the potential of low response rates (esp. in on-line processes) and molding opinions. In our study, more than 50% of the invited experts had participated in the process, which strengthens the validity of its results.

One specific weakness of this study is that the combination of empirical enquiry and normative content matters requires extensive theoretical analysis. As the Delphi approach claims to lead to normative consensus, the results can be taken as such by face value; however, this does not bridge the gap between the ideal and the reality of research.

## 7. Conclusions

The Delphi study was able to augment the thematic spectrum by extending a preliminary list of six needs to a List of Ethical Issues (LEI) of 25 points consented by experts including mental health researchers. This shows that a process of substantial sensibilization was triggered with those involved. This might be possible—and make sense—with larger audiences as well in order to enhance the reflection on ethical issues in research with minors. Moreover, it can be stated that the comprehensive LEI contains items that are largely in accordance with existing guidelines and codes. However, as has been shown previously, not all ethical concerns are covered sufficiently in guidelines as is the case with confidentiality.

Research with minors is ethically conditional, especially due to the vulnerable nature of the population involved; thus, exploring the ethical challenges as well as the interpretation and application of ethical safeguards in mental health research is necessary. From an ethical perspective, the LEI was developed on the basis of the paradigm that acknowledges the competences and supports the rights of minors to participate in the decision-making process, and their ability to make autonomous decisions. This may encourage researchers to adjust their practice of doing mental health research and using the list as a tool for identifying issues to be reflected.

## Figures and Tables

**Table 1:** Preliminary List of Ethical Issues of researchers.

Starting from which age of a research participant should we ask for his/her assent/consent? Starting from which age of a research participant and under what circumstances is obtaining consent from their parents no longer necessary? Should the researcher provide sensitive information to the parents regarding research findings of their offspring? Regarding what age of research participants should we promise to keep the information they provide confidential? Are there circumstances under which we should breach confidentiality with a minor over 16 (giving information to parents, social workers, other authorities)? What should researchers do with participants who are, according to the research findings, “at risk”?

The terminology quoted here is the authentic wording used by the research participants.

**Table 2:** List of Ethical Issues of researchers (reviewed).

**New item**
What special considerations need to be taken into account when obtaining consent in cases with separated parents or children/adolescents in foster care or if parents and minors disagree about the participation in research?
**Item 3**: What level of detailed research findings should be provided to parents regarding their offspring?
**Item 5**: Should the participants be informed prior to the research that confidentiality might be breached in certain circumstances and to whom the information provided might be disclosed (parents, social workers, other authorities?

The new item added and the items 3 and 5 modified.

**Table 3:** Categories.

Assent Consent of a competent minor Circumstances under which the consent of a competent minor might suffice alone Nature of information that should be reported in case of a minor enrolled in a risk-taking behavior study Obligation to disclose Breaching confidentiality with a minor deemed competent to consent Handling of critical data (e.g., self-damaging behavior) if minor decides to withdraw from the study Managing confidential information regarding critical data with minor who reaches the legal age of consent while being enrolled in the study Waiving of parental consent—procedures recommended in a situation in which a minor is competent to give consent, but refuses his/her parents to be asked for their consent

## Abbreviations

ASD	Autism Spectrum Disorder
BPS	British Psychological Society
CIOMS	Council of International Organizations of Medical Sciences
CFR	Code of Federal Regulation
IRB	Institutional Review Board
MHR	Mental Health Research
RCT	Randomized Controlled Trial
WHO	World Health Organization

## Appendix

**Table A1:** List of Ethical Issue (LEI).

CATEGORIES	ITEMS of EACH CATEGORY	Consensus for Acceptation (≤70%)	Consensus for Rejection (≤30%)	Ambiguos Needs (<70% and >30%)
**Assent**	Minor’s assent must be obtained always in addition with parental consent	**80.64%**		
	Assent should contain general information about the purpose of the study and address the information in a language appropriate to the minor’s cognitive development	**96.77%**		
	Minor should be able to understand what is proposed in the study and that he/she has the opportunity to refuse	**100%**		
**Minor consent**	Mature minors can give consent for participating in research if they are deemed to be competent	**87%**		
	The competence requested for the validity of a minor’s consent in research is related to the minor’s cognitive development to understand the information about the nature and purpose of the study, to weigh the information and foresee the consequences, and to arrive at a decision in question	**90.32%**		
	Emancipated minors who are pregnant, parents themselves, in military service or self-supported can give consent for participating in research	**80.64%**		
	The competent minor should show understanding regarding the nature and purpose of the study and consequences of his participation (potential risks, anticipated benefits, and limits of confidentiality)	**100%**		
	The minor should be able to give reasons why he/she wants to participate (in order to justify a reasonable and free participation)	**83.87%**		
	The minor must give consent voluntarily	**96.74%**		
**Circumstances under which the consent of a competent minor might suffice alone**	If the minor is over 16 and the research involves no more than minimal risk or if the risk of harm is potentially low (e.g., questionnaire studies), with IRB approval	**83.87%**		
	If parents withhold their consent to a study with potential major benefit for the minor and if supported by the decision of an Institutional Review Board			**61.29%**
	If the minor is competent to make the decision and if the consequences do not affect the parents or if the issue is not of a child protection	**77.41%**		
	If parents are not involved in the education of the child at present or if they are not available			**51.61%**
	In research involving minors under 16, it is hardly acceptable to take the minor's consent as sufficient alone			**64.51%**
	The minor’s consent cannot be accepted as sufficient alone for research projects. It can be accepted only in practice, for treatment		**16.12%**	
**Nature of information that should be reported in case of a minor enrolled in a risk-taking behavior study**	If there are any major and acute risks of harm to self (suicidal ideation and behavior), pathological gambling, drug abuse, promiscuity	**87.09%**		
	If the minor shows behavior that puts others at serious risk (e.g., planned crime)	**93.54%**		
**Obligation to disclose**	Information should be provided to the parents regarding the overall results of the study in the respective school or group of their child			**61.29%**
	If the child is reporting significant risk behaviors, then direct permission should be sought from the parents and child for a further clinical interview to confirm the issues	**83.87%**		
	If information collected for the research makes researchers believe that medical care is needed for the minor, parents/guardians should be advised to seek medical care	**90.32%**		
**Breaching confidentiality with a minor deemed competent to give consent**	If there is a high risk of harmful behavior to self (suicidal thoughts and ideation, sexual abuse), if the physical or mental health of the minor is in serious danger (severe mental disorder, nocuous drug abuse), if his/her behavior is an acute threat to others	**96.77%**		
	If data show a risk of self-harm and if the child is not compliant to a proposed intervention (e.g., he/she does not come to the therapy sessions)	**70.96%**		
	Information that might be disclosed is if the minor is in need for medical care	**74.19%**		
**Handling of critical data (e.g. self-damaging behavior) if minor decides to withdraw from the study**	Is depending on the minor’s decision			**32.25%**
	Withdrawing does not allow using data			**54.83%**
	It should not be used to calculate any results. All data from the withdrawers should be separated from the active data to analyze the active dropouts using the approach “intention-to-treat“			**64.51%**
	The data should be used anonymously if scientifically possible, but otherwise destroyed			**61.29%**
	It should be deleted if the participant wants and data is retractable	**80.64%**		
	The critical data should be irreversibly deleted			**35.48%**
	Data must be archived and can be used			**41.93%**
**Managing confidential information regarding critical data with minor who reaches the legal age of consent (while being enrolled in the study)**	The management of confidentiality regarding critical data should be part and regulated in consent and assent, prior to the onset of the research.	**87.09%**		
	In studies investigating current suicidal behavior, the results must be immediately checked and clinicians have to be informed right away. Additionally, the minors and their parents should receive contact addresses via the study information about the appropriate help centers, where they can call if any psychological problems or uncertainties concerning health status might arise in the process or as a consequence of the process of the testing	**87.09%**		
	If there is evidence or indication for critical behavior that matches the specified circumstances for breaching confidentiality the parents should be informed	**70.96%**		
	Once the minors are over 18, the information is now theirs and their permission would need to be sought before it was released to a third party	**87.96%**		
**Waiving of parental consent - procedures recommended in a situation in which a competent minor who can give consent, refuses that the parents be asked to give their consent**	An independent expert should be involved to answer the question if the minor is competent to give or refuse consent			**45.16%**
	If the research entails only minimal risks, the adolescents (over age of 14) should be enrolled without parental consent; if risks are higher, it can only be done after the parents consented. Otherwise the minor should not be recruited			**45.16%**
	The minor should be able to justify his/her wish not to inform the parents. If there are still good reasons *(i.e*., parental violence or drug abuse) after discussion and he/she fulfills all criteria for competent consent, he/she should be allowed to participate	**74.19%**		
	The minor should be included in the study if the parents are not affected by the consequences			**32.25%**
	If the study has major implications for the child’s health or life (e.g., self-damage behaviors identification) the involvement of the parents should be included in the consent form as a possibility/necessity under certain conditions. If the child does not want to involve the parents, he/she could be asked about another adult (relative) he/she trusts to (with contact data)			**61.29%**
